# Co-application of Mycorrhiza and methyl jasmonate regulates morpho-physiological and antioxidant responses of *Crocus sativus* (Saffron) under salinity stress conditions

**DOI:** 10.1038/s41598-023-34359-6

**Published:** 2023-05-06

**Authors:** Mohammad Hamidian, Mohsen Movahhedi-Dehnavi, R. Z. Sayyed, Waleed Hassan Almalki, Abdul Gafur, Bahman Fazeli-Nasab

**Affiliations:** 1 Department of Agronomy and Plant Breeding, Faculty of Agriculture, Yasouj University, Yasouj, Iran; 2 Department of Microbiology, PSGVP Mandal’s S I Patil Arts, G B Patel Science and STKV Sangh Commerce College, Shahada, 425409 India; 3 Department of Pharmacology, College of Pharmacy, Umm Al-Qura University, Makkah, 24382 Saudi Arabia; 4 Sinarmas Forestry Corporate Research and Development, Perawang, Indonesia; 5 Department of Agronomy and Plant Breeding, Agriculture Institute, Research Institute of Zabol, Zabol, Iran; 6 Plant Biotechnology and Breeding Department, College of Agriculture, Ferdowsi University of Mashhad, Mashhad, Iran

**Keywords:** Microbiology, Plant sciences

## Abstract

Salinity stress is the second most devastating abiotic factor limiting plant growth and yields. Climate changes have significantly increased salinity levels of soil. Besides improving the physiological responses under stress conditions, jasmonates modulate Mycorrhiza—Plant relationships. The present study aimed to evaluate the effects of methyl jasmonate (MeJ) and *Funneliformis mosseae* (Arbuscular mycorrhizal (AM) on morphology and improving antioxidant mechanisms in *Crocus sativus* L. under salinity stress. After inoculation with AM, pre-treated *C. sativus* corms with MeJ were grown under low, moderate, and severe salinity stress. Intense salinity levels damaged the corm, root, total leaf dry weight, and area. Salinities up to 50 mM increased Proline content and Polyphenol oxidase (PPO) activity, but MeJ increased this trend in proline. Generally, MeJ increased anthocyanins, total soluble sugars, and PPO. Total chlorophyll and superoxide dismutase (SOD) activity increased by salinity. The maximum catalase and SOD activities in + MeJ + AM were 50 and 125 mM, respectively, and the maximum total chlorophyll in –MeJ + AM treatment was 75 mM. Although 20 and 50 mM increased plant growth, using mycorrhiza and jasmonate enhanced this trend. Moreover, these treatments reduced the damage of 75 and 100 mM salinity stress. Using MeJ and AM can improve the growth of saffron under various ranges of salinity stress levels; however, in severe levels like 120 mM, this phytohormone and *F. mosseae* effects on saffron could be adverse.

## Introduction

*Crocus sativus* L., commonly known as saffron, is an economically vital medicinal and aromatic plant known as the Golden Condiment. It is the world's highest-priced spice derived from its dry stigmas. The major components of *Crocus sativus* stigma are the saponins, crocin, crocetin, and safranal. Saffron has numerous medicinal and nutritional uses. Saffron enhances the antioxidant capacity, acts as a free radical scavenger, and modulates inflammatory mediators and immune responses^1–3^.

Estimations showed that 830 million hectares of land worldwide are under salinity stress and increase annually^4–6^. It can be a threat to agricultural products around the globe^7–9^. Although there is little research about the effect of salinity stress on Saffron (*Crocus sativus* L.), its adverse effects have been partly observed on it^3^. However, the deficiency of reports on the impact of salinity stress on morpho-physiological responses of this valuable medicinal plant is much felt. *C. sativus* is one of the few crops of the Iridaceae family. The dried red stigmas of that are known as the most expensive spice in the world; hence, it has been named red gold. Also, this plant has a particular morphology with several different underground organs and a particular leafing type. Indeed, saffron can produce other by-products besides their original yields, such as stamens, styles, and corms, which are valuable in some industries. Therefore, the plant morphological organs resulted in supplementary incoming and increasing profitability in saffron farms^4,10^. Due to its coloring, flavoring, and perfume potential related to primary metabolites (crocin, picrocrocin, and safranal), this spice is widely used in food and alcoholic beverages^11^. Besides its food industry usage, it has medical properties like antidepressants, anticancer, anti-inflammatory, and antioxidant activity^12^.

Salinity restricts plant growth by creating osmotic potential in the root environment (physiological drought), disturbing nutrient hemostasis, ion toxicity, and producing reactive oxygen species (ROS). At the same time, disruption of cell membrane structures, the photosynthesis system disorder, and even cell death are the negative consequences of increasing ROSs under salinity^2^. To combat salinity stress, plants undergo biochemical, physiological, and molecular changes^13^. For example, increasing osmolytes (such as soluble sugars and proline) occur to regulate osmotic status, ion balance, and mineral homeostasis in plants^14^. Moreover, increasing the activity of enzymatic and non-enzymatic antioxidants reduces the damage related to ROS in salinity stress^15^. In general, the control of these physiological and biochemical processes is based on the stimulated expression of genes in which phytohormones play an essential role^16^.

Abiotic stress stimulates jasmonate biosynthesis, increasing the production of enzymatic defenses by promoting related mRNA^17^. Thus, jasmonates can reduce salinity damage by improving the antioxidant power of plants^18^. In recent years, exogenous applications of methyl jasmonate have been used to reduce salinity damage in chamomile^19^, soybean^13^, basil^20^, and rapeseed^21^. In all these research studies, jasmonate application has reduced salinity stress damage due to improving plant antioxidant defense and osmoregulation. Furthermore, another beneficial effect of jasmonates under salinity stress conditions is related to synthesizing non-enzymatic antioxidants (such as anthocyanins) by regulating the phenylpropanoid pathway^22^.

Arbuscular mycorrhizal (AM) improve plant growth under stress by increasing nutrient uptakes and water absorption and strengthening the defense system of plants. In addition, increased hormone production in inoculated plants with mycorrhiza reduces salinity damage^23^. It was reported that during mycorrhization, the jasmonates increase endogenously in plants^24^. On the other hand, it was mentioned that increasing jasmonate in mycorrhizal roots led to resistance of plants to biotic^25^ and abiotic stresses^26^. However, exogenous use of methyl jasmonate may reduce plant inoculation with AM^27^.

Jasmonates have a critical role in symbiosis. Less colonization has been observed in plants, which produce less endogenous jasmonate than their wild counterparts^28^. The potential of plants in synthesizing this hormone endogenously and exogenous application could affect the efficiency of mycorrhization^26^. More important than that is the consequence of combining these factors on physiological and developmental reactions in plants under different stresses.

As noted earlier, since the information about the responses of *C. sativus* in salinity stress conditions is insufficient, studying the morpho-physiological responses of this plant under different salinity stress conditions can be beneficial. In addition to that, recently, there has been a growing interest in using external substances to reduce stress damage. From the sustainable agriculture perspective, rhizosphere microorganisms are considered efficient components to reduce stress damage and improve production. Application of mycorrhizae has been reported to enhance *C. sativus* stigma quality and yields^29^. On the other hand, Jasmonates can modulate symbiosis relationships between plants and mycorrhizae^26^. More importantly, evaluating the effect of this phytohormone and mycorrhizal inoculation on plant physiology response. This approach can provide helpful information on the optimal use of these factors in cultivating *C. sativus* as a valuable medicinal plant and spice. The present study aimed to identify the effect of methyl jasmonate on the morpho-physiological responses of *C. sativus* inoculated with *Funneliformis mosseae* under different levels of salinity stress.

## Materials and methods

### Collection and studies on plant material

The collection of *C. sativus* (plant) material was performed according to institutional, national, and international guidelines. Plant studies and all experimental procedures were performed in conformity with applicable institutional, national, and international guidelines. Plants were identified by Sedigheh Khademian, botanist of Shiraz Faculty of Pharmacy Department, Shiraz University of Medical Sciences, Shiraz, Iran (Herbarium number: PM1427- *Crocus sativus* L.).

*C. sativus* corms (cultivated form) were collected from a two-year-old field from Agricultural Jihad Management Organization, Torbat-e Heydariyeh, Razavi Khorasan Province, Iran (35° 25′ 77" N 59° 22′ 91" E). The healthy and uniformed corms were carefully selected with an average size of 7 g for cultivation because the growth and yield of *C. sativus* are highly dependent on the size of the primary corms.

The city of Torbat-e Heydariyeh has an area of 53 square kilometers. Its height is 1333 m above sea level. Torbat-e Heydariyeh region is considered the largest producer of saffron in the world. The area under saffron cultivation in Torbat-e Heydariyeh is 8000–9000 hectares, and about 35–40 tons of dry saffron are produced annually.

Since the species in question is widely distributed throughout the country, no certificate or permission was required to gather the samples; however, the dean of the Agricultural Jihad Management Organization (Torbat-e Heydariyeh) was consulted before sampling.

### Experimental design and treatments

The experiment was conducted in pots in the research greenhouse of Yasouj University in a completely randomized factorial design with three replications. The first factor included 6 salinity stress levels irrigated in 25, 50, 75, 100, and 125 mM NaCl with a control treatment (modified Hoagland nutrient solution for mycorrhization)^30^. The second factor included arbuscular mycorrhizal fungus inoculation (*Funneliformis mosseae*) without fungal application. The third factor was the pre-treatment use of 75 µM methyl jasmonate and its non-use.

### Preparation of pots and treatment applications

The culture medium of *C. sativus* was perlite^31^ (4–5 mm), and five corms were planted with a depth of 15 cm in UV plastic pots with a height of 30 and a diameter of 20 cm. The perlite was washed and disinfected in an autoclave before the test. A complete Hoagland solution with pH 5.6 and 2.5 dS was used for irrigating pots. Corms were soaked in 75 µM methyl jasmonate solution for 24 h, and the control treatment was soaked in distilled water. Each pot with 450 CC of Hoagland solution was irrigated by applying different stress treatments from the first irrigation. For better inoculation and prevention of mycorrhizal spores removal from the substrate during irrigation, the fungal spores were mixed with fine-grained perlite (2–3 mm) and coco peat (3:1). Perlite, coco peat, and spores were placed 5 cm layer below the corms. Also, for non-inoculated treatments same materials without mycorrhizal spores were used.

### Sampling for the physiological traits

Leaves were randomly sampled from 2 plants per pot to measure physiological traits 120 days after salinity stress. Leaf samples were placed in a liquid nitrogen container, transferred to the laboratory, and stored at − 40 °C since to be used. Three plants took from each pot to measure the total dry weight of the plant and the leaf area. In addition, the different components of the plant were dried separately at 70 °C for 72 h after measuring the leaf area. Then, the dry weight was calculated by a scale.

### Measurement of colonization and mycorrhizal dependency (MD) index

The fresh root samples, 1 cm long, in KOH (10%), were de-stained in the Ben Mary to calculate colonization in mycorrhizal fungus treatments. The 5% blue ink in acetic acid was used for de-staining^32^. After de-staining, the percentage of colonization was calculated^33^. MD index was obtained based on the following equation^34^.

$$\mathrm{MD }(\mathrm{\%})=\frac{\mathrm{DW of mycorrhizal plants }-\mathrm{DW of Non}-\mathrm{mycorrhizal plants}}{\mathrm{DW of mycorrhizal plants}}\times 100$$

### Measurement of leaf chlorophyll and carotenoid content

Fresh leaf samples (1 g) were homogenized in low light in 80% cold acetone with calcium carbonate powder. Following the centrifugation at low temperature, the absorption of the solution was read at 663, 645, and 470 nm using a spectrophotometer (Lambda 210 EZ)^35^.

### Leaf anthocyanin content

The leaf sample (0.2 g) was homogenized in acidic methanol (HCl: Methanol, 1:99, v/v). The absorption spectra of the extracts were determined by a spectrophotometer (Lambda 210 EZ) at a wavelength of 530 nm^36^.

### Preparation of enzymatic extract

The extraction buffer (potassium phosphate pH = 7.8, EDTA, and PVP) was homogenized with 0.1 g of the leaf sample in a mortar at a low temperature. Then, the samples were centrifuged, and the supernatant was used to measure enzyme activity^37^.

### Leaf catalase activity

CAT activity was measured at 240. It was based on reducing hydrogen peroxide absorption in the reaction mixture (50 mM phosphate buffer containing 30 mM oxygenated water and 100 μL of enzyme extract in pH = 7). The reaction was started by adding H_2_O_2,_ and the adsorption decreased for 60 s. The extinction coefficient for CAT was 0.0394 mmol^−1^ cm^−1^^38^.

### Leaf polyphenol oxidase activity

PPO activity was measured at 420 nm. It was based on the increase of enzyme activity on the intensity of orange dye of the produced methyl catechol in the reaction mixture (100 μL of enzyme extract, 500 μL of 5 mM oxygenated water, and 500 μL of 0.02 mmol methyl catechol in 1900 μL of the 60 mM phosphate buffer with pH = 1.6)^39^.

### Leaf superoxide dismutase activity

SOD activity was measured based on the inhibition of nitroblutotrazolium (NBT) under light conditions created with the fluorescent lamp at 25 °C. The solution was measured by a spectrophotometer (Lambda 210 EZ) at 560 nm. Its activity was determined based on the difference between the read solution and the NBT complete reduction conditions^40^.

### Leaf proline content

Leaf proline content was measured^41^ using the alcoholic extract method (1 g leaf fresh weight in 15 cc ethanol). Double distilled water, ninhydrin solution, and glacial acetic acid were added to measure the proline content of the alcoholic extract. Then, benzene was added after placing the samples in the patient to enter the benzene phase. Proline content was measured based on the light absorption of the samples at 515 nm using a spectrophotometer.

### Total leaf soluble sugars content (TSS)

Alcoholic extracts (1 g leaf fresh weight in 15 cc ethanol) and anthrones were placed in the Ben Mary, and the absorbance of the samples was read at 625 nm using a spectrophotometer after cooling the samples in laboratory^42^. l-proline and methyl jasmonate from Sigma-Aldrich and other chemicals were prepared from Merck in this test.

### Statistical analysis

LSD test was used to compare the means of the main effects and the interaction. The ANOVA was performed by SAS ver. 9.1 and graphs were plotted by Excel 2016 software.

### Ethics approval and consent to participate

No humans or animals were used in the present research.

### Informed consent

No human volunteers were involved in this research.

### Statement of plant guidelines

The collection of *C. sativus* (plant) material was performed according to institutional, national, and international guidelines. Plant studies and all experimental procedures were performed in conformity with applicable institutional, national, and international guidelines. Plants were identified by Sedigheh Khademian, botanist of Shiraz Faculty of Pharmacy Department, Shiraz University of Medical Sciences, Shiraz, Iran (Herbarium number: PM1427- *Crocus sativus* L.).

## Results

### Effect of methyl jasmonate and salinity stress on root colonization and MD index

**Figure 1:**
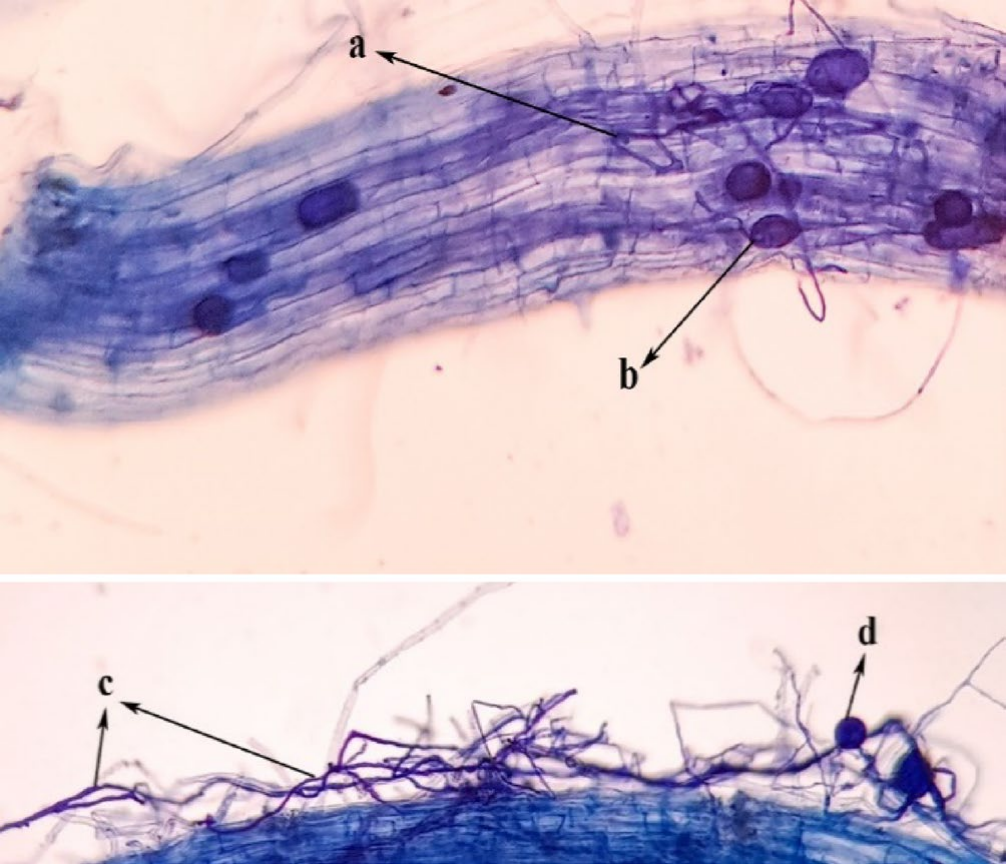
Colonization of *F. mosseae in C. sativus* roots (**a**) internal hyphae, (**b**) vesicles, (**c**) external hyphae, and (**d**) spore.

Internal hyphae, vesicles, external hyphae, and spores, as fungal bodies, are represented in Fig. 1. Salinity and MeJ interaction was significant in both traits (Table 1). Increased salinity reduced root colonization in + MeJ and − MeJ treatments. However, the MD index had an increasing trend compared to the control in MeJ treatments with increasing salinity levels. In fact, in this treatment, the difference in dry weight of plants in + AM than − AM treatments was increased with increasing salinity stress.

**Table 1:** Variance analysis and mean comparison for the interaction of salinity and MeJ on MD and colonization.

Salinity	MeJ	MD%	Colonization%
Control	− MeJ	16.44^c^	74.0^c^
25 mM	− MeJ	18.87^bc^	70.7^c^
50 mM	− MeJ	16.22^c^	63.0^d^
75 mM	− MeJ	21.25^abc^	47.3^e^
100 mM	− MeJ	31.31^a^	28.0^g^
125 mM	− MeJ	23.59^abc^	28.3^g^
Control	+ MeJ	30.24^ab^	86.5^a^
25 mM	+ MeJ	26.24^abc^	79.7^b^
50 mM	+ MeJ	19.35^bc^	63.6^d^
75 mM	+ MeJ	20.28^abc^	35.7^f^
100 mM	+ MeJ	19.83^abc^	28.5^g^
125 mM	+ MeJ	− 19.19^d^	22.3^h^
S.O.V	df		
Salinity	5	**	**
MeJ	1	*	ns
Salinity × MeJ	5	**	*

Mean values with the same letters show no significant difference based on the LSD test.

*, **, and ns present significance at 5 and 1% probability levels and non-significant, respectively.

In + MeJ treatments, root colonization, and MD were higher at slight salinity among other stress levels; However, the maximum amount of both traits were in + MeJ treatment in the non-stress condition. The minimum root colonization percentage was in plants treated with + MeJ under 125 mM stress level. Nevertheless, it was significant only at a 100 mM level. Also, the amount of MD index was negative in + MeJ treatment under 125 mM stress. This means treatments reduced the plant's dry weight by 19.2% compared to non-treated plants (Table 1).

### Effect of AM colonization, methyl jasmonate, and salinity stress on leaf chlorophyll and carotenoid content and chlorophyll fluorescence

The three-way interaction of salinity, mycorrhiza, and jasmonate significantly affected total chlorophyll (Chl) and leaf Chl b content. However, none of the applied treatments on Fv/Fm changes were significant (Table 2). The amount of Chl b and total Chl increased by increasing salinity up to 75 mM, then decreased. However, Table 3 shows that in almost all treatments, Chl b and total Chl under salinity stress were higher than in non-stress conditions. The maximum levels of Chl b and total Chl were in – MeJ − AM and + MeJ − AM under 75 mM stress. Chl increased to 75 mM salinity and then decreased to 100 and 125 mM (Table 3). Treatment + MeJ + AM strongly affected Chl b synthesis under 25 and 50 salinity, but after that, this trend changed by increasing stress levels. Also, in the + MeJ + AM treatment, the total Chl content at 25 and 50 mM salinity levels were at least 43% and 27% higher than other treatments at the same stress level, respectively. In − MeJ + AM treatment, Chl b and total Chl increased significantly up to 50 mM. Then its value stayed constant. Therefore, none of the salinity levels significantly differed from this level after that in terms of total Chl and Chl b (Table 3).

**Table 2:** Variance analysis of the effects of salinity, MeJ, and AM on physiological traits of *C. sativus.*

S.O.V	df	Chla	Chlb	Chl a + b	Carotenoid	FV/FM	Anthocyanin	PPO	SOD	CAT	Proline	TSS
Salinity	5	ns	**	**	**	ns	**	**	**	**	**	ns
MeJ	1	ns	**	**	**	ns	*	*	**	ns	**	*
AM	1	ns	ns	ns	ns	ns	ns	*	**	ns	ns	ns
Salinity × MeJ	5	ns	ns	ns	**	ns	ns	ns	**	**	*	ns
Salinity × AM	5	ns	**	**	**	ns	*	ns	**	**	**	ns
AM × MeJ	1	ns	ns	ns	**	ns	ns	ns	**	ns	**	ns
Salinity × AM × MeJ	5	ns	**	*	**	ns	ns	ns	**	**	**	ns

*AM* arbuscular ycorrhizal, *MeJ* methyl jasmonate.

*, **, and ns present significance at 5 and 1% probability levels and non-significant, respectively.

**Table 3:** Mean comparison of the interaction of salinity, MeJ, and AM for leaf chlorophyll a and b and carotenoids content.

Salinity	MeJ	AM	Chlb (mg g^−1^_FW_)	Chla + b (mg g^−1^_FW_)	Carotenoid (mg g^−1^_FW_)
Control	− MeJ	− AM	0.236^ef^	1.048^i^	0.0181^h^
	− MeJ	+ AM	0.148^f^	1.136^hi^	0.0210^gh^
	+ MeJ	− AM	0.411^de^	1.288^ghi^	0.0289^bcde^
	+ MeJ	+ AM	0.327^def^	1.363^efghi^	0.0280^cdef^
25 mM	− MeJ	− AM	0.258^ef^	1.120^hi^	0.0206^gh^
	− MeJ	+ AM	0.252^ef^	1.160^hi^	0.0300^abcd^
	+ MeJ	− AM	0.262^ef^	1.111^i^	0.0305^abc^
	+ MeJ	+ AM	0.701^bc^	1.656^cdef^	0.0277^cdef^
50 mM	− MeJ	− AM	0.707^bc^	1.521^cdefg^	0.0237^fg^
	− MeJ	+ AM	0.841^b^	1.547^cdefg^	0.0276^cdef^
	+ MeJ	− AM	0.773^b^	1.609^cdefg^	0.0321^abc^
	+ MeJ	+ AM	1.269^a^	2.053^ab^	0.0294^abcd^
75 mM	− MeJ	− AM	1.344^a^	2.176^a^	0.0288^bcde^
	− MeJ	+ AM	0.634^bc^	1.323^fghi^	0.0276^cdef^
	+ MeJ	− AM	1.379^a^	2.210^a^	0.0330^ab^
	+ MeJ	+ AM	0.697^bc^	1.725^cd^	0.0339^a^
100 mM	− MeJ	− AM	0.498^cd^	1.496^defg^	0.0316^abc^
	− MeJ	+ AM	0.849^b^	1.707^cde^	0.0317^abc^
	+ MeJ	− AM	0.806^b^	1.726^cd^	0.0316^abc^
	+ MeJ	+ AM	0.720^b^	1.449^defgh^	0.0331^ab^
125 mM	− MeJ	− AM	0.732^b^	1.691^cde^	0.0245^efg^
	− MeJ	+ AM	0.698^bc^	1.530^cdefg^	0.0340^a^
	+ MeJ	− AM	0.814^b^	1.843^bc^	0.0317^abc^
	+ MeJ	+ AM	0.780^b^	1.584^cdefg^	0.0252^defg^

Mean values with the same letters show no significant difference based on the LSD test.

*AM* arbuscular mycorrhizal, *MeJ* methyl jasmonate.

Different letters indicate significant differences.

The three-way interaction of salinity, mycorrhizal, and jasmonate on leaf carotenoid content The three-way interaction of salinity, mycorrhiza, and jasmonate on leaf carotenoid content was significant (Table 2). Leaf carotenoid content increased by increasing salinity stress levels, but this upward trend in – MeJ − AM and + MeJ + AM treatments was till 100 mM. Pre-treatment with MeJ influenced the carotenoid content, particularly in the control and slight and moderate stress conditions. Indeed, carotenoid content in + MeJ (in both + AM and − AM treatments) was significantly higher than – MeJ − AM under 25, 50, and 75 mM stress levels (Table 3). The maximum carotenoid content was observed in + MeJ + AM and − MeJ + AM treatments at 125 mM and 75 mM salinity levels, respectively (Table 3).

### Effect of AM colonization, methyl jasmonate, and salinity stress on leaf anthocyanin content

**Figure 2:**
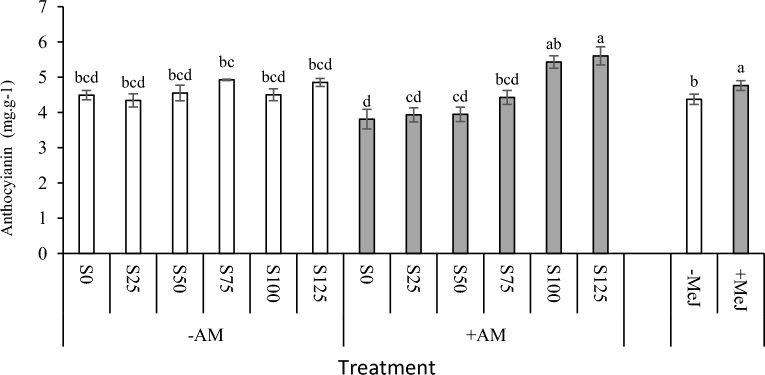
Interaction of salinity and AM and the main effect of MeJ on leaf anthocyanin content. S0 = no salinity, S25 = 25 mM NaCl, S50 = 50 mM NaCl, S75 = 75 mM NaCl, S100 = 100 mM NaCl, S125 = 125 mM NaCl. *AM* arbuscular mycorrhizal, *MeJ* methyl jasmonate. Columns with the same letters are not significantly different based on LSD. Mean ± standard error.

The results showed a significant two-way interaction between salinity and mycorrhiza and the main effect of MeJ on anthocyanin content (Table 2). Anthocyanin content increased by increasing salinity stress levels. Still, the anthocyanin content in non-inoculated plants with mycorrhiza was insignificant in salinity levels. The anthocyanin amount increased by increasing salinity levels in inoculated plants by mycorrhizae. The maximum amount was related to + AM treatment under 125 mM stress, significantly higher than all stress levels. Generally, plants pre-treated with MeJ had significantly higher anthocyanin levels than − MeJ (Fig. 2).

### Effect of AM colonization, methyl jasmonate, and salinity stress on leaf enzymatic antioxidants

**Figure 3:**
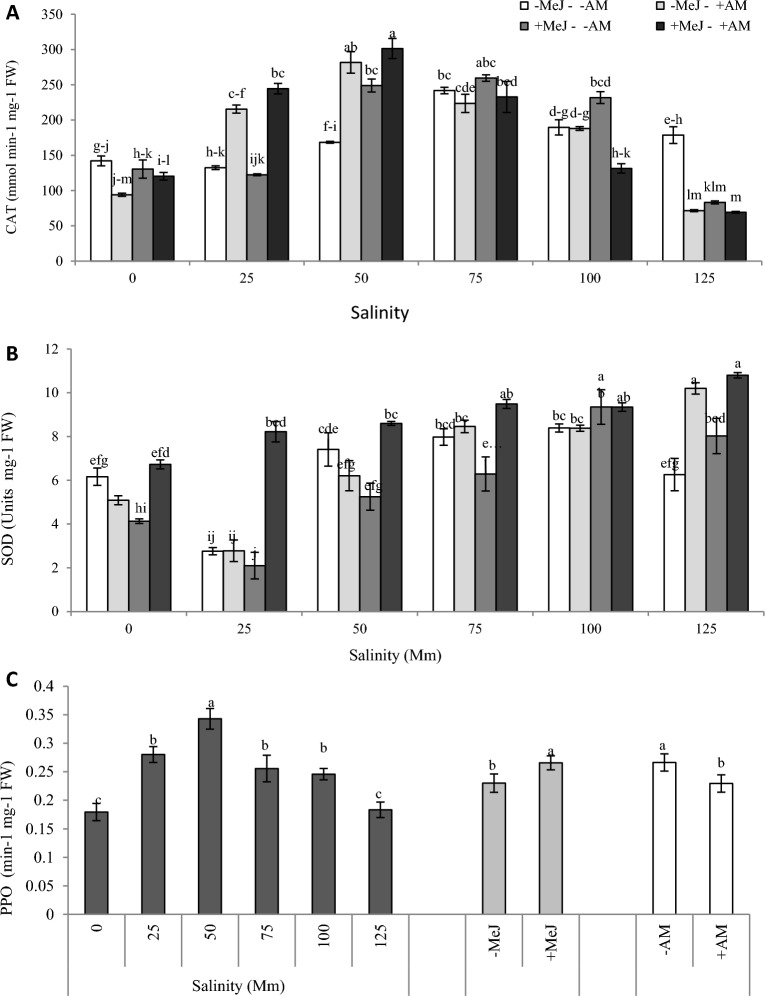
Interaction of salinity, AM, and MeJ on leaf CAT (**A**) and SOD (**B**) activity. (**C**) shows the main effects of salinity, AM, and MeJ on PPO activity. *AM* arbuscular mycorrhizal, *MeJ* methyl jasmonate. Columns with the same letters are not significantly different based on LSD. Mean ± standard error.

The three-way interaction of experimental factors on SOD and CAT enzyme activity was significant (Table 2), but only the main effects were significant on PPO activity (Table 2). Salinity stress increased the CAT activity to a certain level of salinity, which differed in each treatment. Then CAT activity decreased in severe stress conditions. The activity of CAT decreased in all treatments at extreme stress levels (100 and 125 mM). In – MeJ − AM and + MeJ − AM treatment, this incremental trend was up to 75 mM salinity level (Fig. 3A). However, the gradual trend of CAT activity in − MeJ + AM and + MeJ + AM was up to 50 mM, significantly higher than other stress levels. However, the incremental trend of CAT activity in − MeJ + AM and + MeJ + AM was up to 50 mM, significantly higher than other stress levels. CAT activity in pre-treated plants with MeJ was significantly higher than MeJ −AM in low salinity stress levels (25 and 50 mM).

SOD activity in – MeJ − AM, +MeJ −AM, and − MeJ + AM treatments under 25 mM salinity was significantly decreased compared to non-stressed conditions. Then, it increased with increasing salinity from 50 mM (Fig. 3B). Nevertheless, the activity of this enzyme in these treatments under 125 mM stress was less than − MeJ − AM (Fig. 3A). + MeJ − AM treatment reduced the SOD activity significantly under 75, 50 mM stress, and control condition. While at + MeJ treatments under 125 mM stress, the SOD activity reached the highest value. In general, + MeJ + AM treatment increased SOD enzyme activity more than the control treatment at all stress levels, but it was significant only at salinity levels of 125 and 25 mM.

The saline irrigation water caused a significant increase in the PPO activity of plants up to 50 mM stress level, then decreased at higher levels. However, the PPO activity under all salinity levels was higher than the control. Furthermore, the + MeJ + AM treatment had higher SOD activity compared to + MeJ − AM and − MeJ + AM treatments at all salinity levels (Fig. 3B). Inoculating mycorrhizal fungi causes a significant reduction in the activity SOD. In contrast, more enzyme activity was recorded in + MeJ treatments (Fig. 3C).

### Effect of AM colonization, methyl jasmonate, and salinity stress on the total soluble sugars and leaf soluble proline content

**Figure 4:**
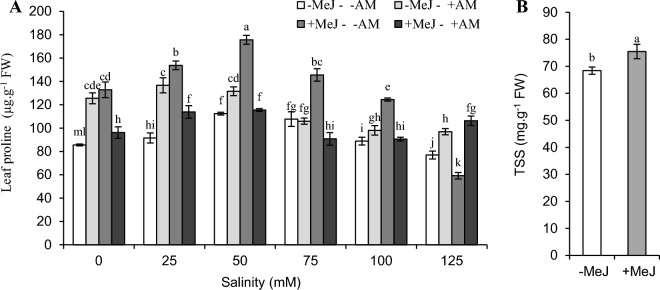
Interaction of salinity, AM, and MeJ on leaf proline content (**A**). (**B**) The main effect of MeJ on leaf total soluble sugars activity. *AM* arbuscular mycorrhizal, *MeJ* methyl jasmonate. Columns with the same letters are not significantly different based on LSD. Mean ± standard error.

The effect of salinity stress, mycorrhizal fungi, and their interaction on the content of TSS was not significant, and only the main effect of MeJ was significant (Table 2). The mean comparison showed that MeJ increased TSS by 10% compared to − MeJ (Fig. 4B). The three-way interaction of salinity, mycorrhizae, and MeJ on leaf proline content was significant. In almost all treatments under 25 and 50 mM salinity stress, leaf proline increased, then by increasing salinity level, this osmolyte decreased. A decrease in proline was observed in almost all treatments at 75, 100, and 125 mM stress levels. Alternatively, the highest proline content at 50, 75, and 100 mM stress was related to + MeJ − AM treatment. However, a sharp decrease in proline content was observed under 125 mM stress in this treatment. Therefore, the minimum proline content was in the + MeJ − AM treatment and was significantly lower than all treatments. Although the proline decrease occurred at stress levels of 75, 100, and 125, its amount was insignificant in + AM. In other words, under these stress levels, the amount of proline remained constant in the inoculated plants with mycorrhiza in treated and non-treated MeJ (Fig. 4A).

### Effect of AM colonization, methyl jasmonate, and salinity stress on main, secondary, and total leaf area index

**Figure 5:**
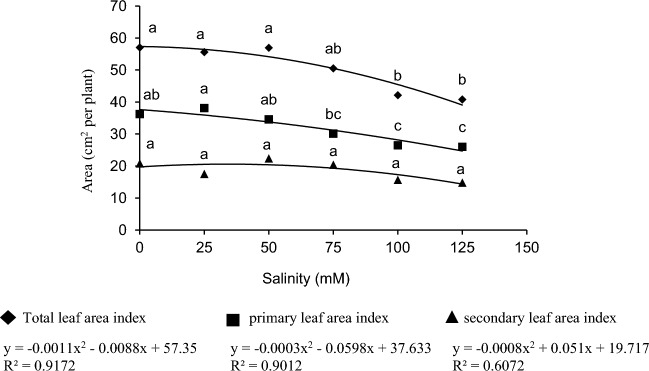
Effect of salinity on primary, secondary, and total leaf area. Mean values with the same letters are not significantly different based on LSD.

The first leaves of *C. sativus* appear from the central bud in the center of the mother corm. Secondary leaves are formed by the growth of lateral buds on the mother corm, which grows in number and area of leaves more minor than the primary leaves and with a time interval (not much) after the main leaf. According to Fig. 5, the primary leaf forms about 63.5, 68.3, and 60% of the total leaf area of a plant of *C. sativus* under control conditions of 25 and 50 mM salinity, respectively.

The main effect of salinity stress on the leaf area (primary leaf) and total leaf area was significant. Still, none of the applied treatments significantly impacted the secondary leaf area (Table 4). Increased salinity stress decreased the leaf area in all treatments compared to the control. However, the minimum total leaf area was generally related to the 125 mM treatment, 28% (equivalent to 16.22 cm^2^) lower than the control treatment. The maximum main leaf area was linked to the 25 mM treatment, but no significant difference existed between the control and 50 mM treatments. However, increasing salinity generally reduced the main leaf area (Fig. 5).

**Table 4:** Variance analysis of the effect of salinity, AM, and MeJ on different *C. sativus* organs.

SOV	df	Corm	Mother corm	Leaf	Stem sheath	Absorbing root	Contractile root	Total dry weight	Primary leaf area	Secondary leaf area	Total leaf area
Salinity	5	**	ns	**	**	**	ns	**	**	ns	*
MeJ	1	**	ns	ns	ns	ns	ns	**	ns	ns	ns
AM	1	*	ns	ns	ns	ns	ns	*	ns	ns	ns
Salinity × MeJ	5	**	ns	ns	*	ns	ns	**	ns	ns	ns
Salinity × AM	5	*	ns	ns	*	ns	ns	*	ns	ns	ns
AM × MeJ	1	ns	ns	ns	ns	ns	ns	ns	ns	ns	ns
Salinity × AM × MeJ	5	ns	ns	ns	ns	ns	ns	ns	ns	ns	ns

*AM* arbuscular mycorrhizal, *MeJ* methyl jasmonate.

*, **, and ns present significant at 5 and 1% probability levels and non-significant, respectively.

### Effect of AM colonization, methyl jasmonate, and salinity stress on the dry weight of each *C. sativus* organ

**Figure 6:**
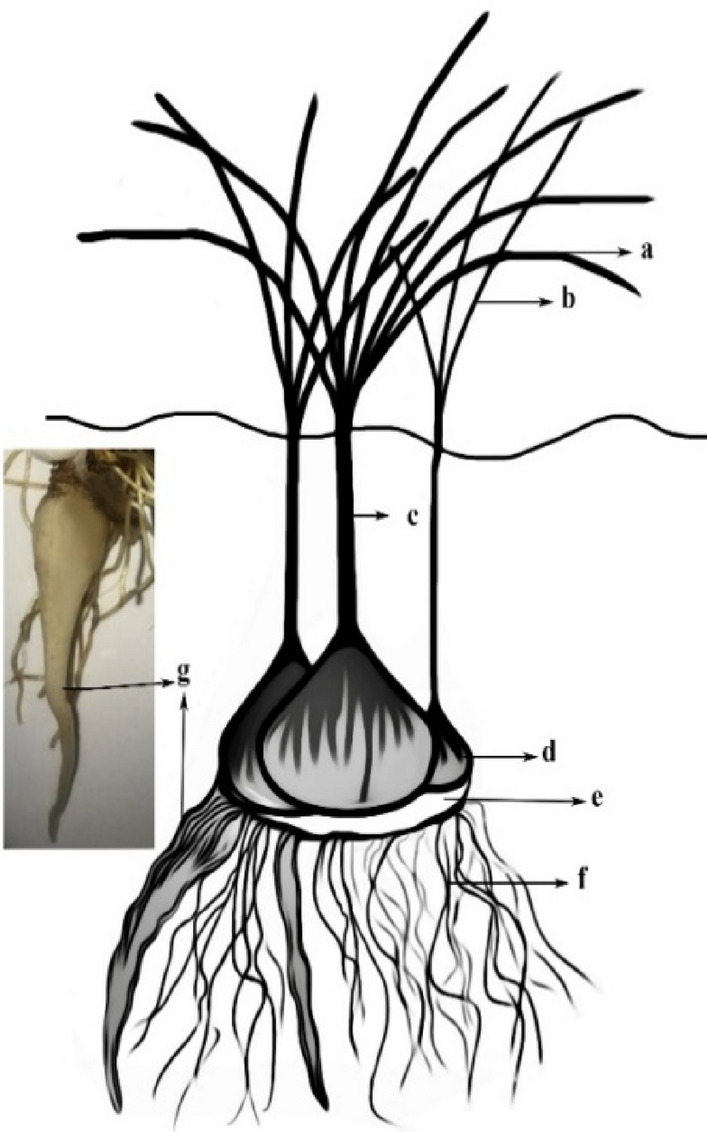
The appearance of *C. sativus* and its components after 120 days of cultivation where (**a**) primary leaf, (**b**) secondary leaf, (**c**) stem sheath, (**d**) daughter corm, (**e**) mother corm, (**f**) absorbing roots and (**g**) contractile roots.

*C. sativus* plants had different parts in the certain growth period (Fig. 6) that were examined separately. According to variance analysis (Table 4), the effect of none of the treatments was significant on the dry weight of the mother corm and contractile root. The two-way effect of mycorrhiza and jasmonate was significant on the corm, stem, and total dry weight (Table 4).

The results of the daughter corm and total dry weight change through a second-degree regression diagram were presented. Besides, first-degree regression diagrams were established to indicate the dry weight of the stem, leaf, and absorbent root. This approach was utilized to understand plants' morphological behavior under salinity stresses. The main effect of salinity stress was significant on the corm, leaf, stem, root, and total dry weight (Table 4). The daughter corm and total dry weight in 25 mM were significantly higher than all components. The dry weight of these components at 50 mM was still considerably higher than the control treatment.

**Figure 7:**
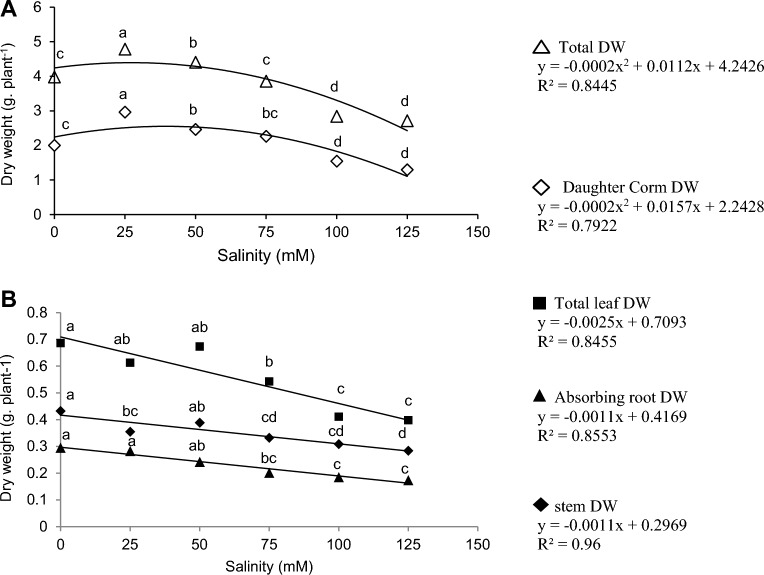
Effect of salinity on (**A**); daughter corm and total dry weight, (**B**); total leaf, absorbing root, stem. Mean values with the same letters are not significantly different based on LSD.

Based on the equation from the regression diagram, the maximum total dry weight (R^2^ = 0.84) and corm (R^2^ = 0.79) were obtained at 28 and 39.2 mM salinity levels. The daughter corm and total dry weight at 75 mM level were not significantly different from the control treatment, but it significantly reduced this factor at 100 and 125 mM. Stem and leaf dry weight at the control level and the two initial levels of 25 and 50 mM stress were not significantly different but decreased significantly at 100 and 125 mM levels. The trend of salinity effect on reducing the dry weight of leaves stems, and roots was a first-order linear graph. The slope of the graphs for leaves stems, and roots were − 0.0025, − 0.0011, and − 0.0011 g dry weight/ mM salinity, respectively (Fig. 7B).

Moreover, the regression coefficient was at least higher than 84% in all graphs. In this way, the minimum amount was related to 125 mM salinity treatment. Salinity stress also reduced root dry weight, but no significant difference was observed at 25, 75, and 100 levels. In addition, the minimum root dry weight was observed at 125 mM (Fig. 7A).

**Figure 8:**
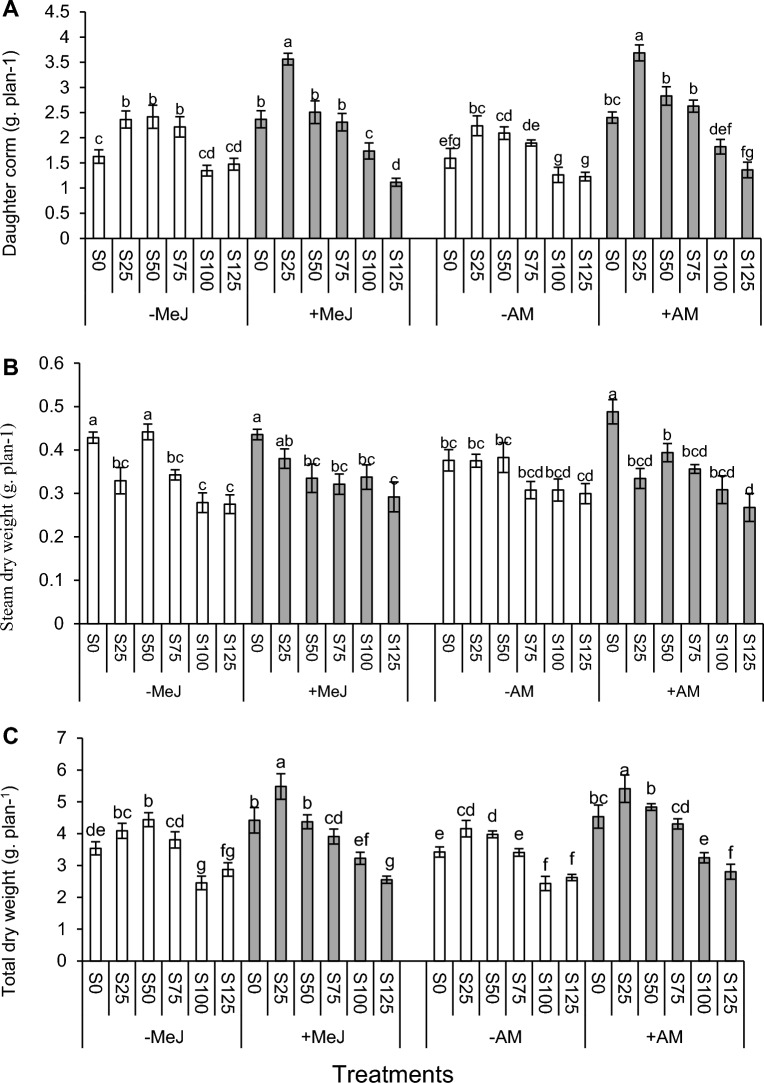
Two-way interaction of AM and salinity and MeJ and salinity on corm (**A**), stem (**B**), and total dry weight (**C**). *AM* arbuscular mycorrhizal, *MeJ* methyl jasmonate. Columns with the same letters are not significantly different based on LSD. Mean ± standard error.

Based on Fig. 8A, in all stress levels except salinity of 125 mM, MeJ increased corm dry weight, but this trend was significant in control and 25 mM stress conditions. Furthermore, inoculation of plants with mycorrhizae increased corm dry weight at all salinity levels. However, a salinity level of 25 mM significantly improved corm dry weight (Fig. 8A). The dry weight of the stem decreased by increasing the salinity stress level. But in pre-treated plants with MeJ, no significant difference was observed between levels 25, 50, 75, and 100 mM. No significant difference was observed in the dry weight of the stem between + MeJ and − MeJ treatments at all salinity levels except 50 mM (Fig. 8B).

Inoculation of plants with mycorrhiza increased total dry weight at all stress levels. Applying jasmonate increased the plant's total dry weight by 34 and 31% at 25 and 100 mM salinity levels, respectively, compared to the control (no application of jasmonate). This increase was insignificant only at 125 mM levels (Fig. 8C).

## Discussion

The MD index shows the percentage of mycorrhizal plant changes in one trait (such as dry weight) compared to non-inoculated plants. Although root colonization significantly reduced in − MeJ treatments under stress in the current study, mycorrhiza positively affected plant growth and development. Indeed, the MD index in − MeJ treatments was higher in severe salinity stress levels compared to slight stress levels and control conditions. Similar to the results of the current study (in − MeJ treatments), other reports have shown an increase in MD% (based on plant dry weight) by increasing salinity levels^34,43^. Mycorrhiza could be more effective for plants under stress conditions by improving enzymatic and non-enzyme antioxidants rather than in non-stress conditions^44^. Maximum MD and colonization were seen in plants pre-treated with MeJ at the control level. However, the MD index decreased by increasing salinity levels in this treatment. Finally, using AM and MeJ adversely affected plant growth under 125 mM stress, so the MD value became negative. It was reported that jasmonate positively and significantly affects plant colonization with mycorrhizal fungi. However, the impact of this hormone depends on the applied concentrations^26^. The concentration of 75 μM under non-stress conditions positively affected plant colonization and growth.

On the other hand, it has been reported that jasmonic acid in saline conditions is synthesized endogenously^45^. The increase in salinity may affect the synthesis of jasmonate endogenously. Therefore, changes in the concentration of this hormone may reach a point that causes damage to mycorrhization and plant growth.

The response of photosynthetic pigments to salinity stress differs based on the stress period. Generally, a decrease in chlorophyll at salinity conditions is expected due to limited stomatal diffusion and destruction of the photosynthetic system^43^. However, it was observed that chlorophyll in salinity stress has increased in slight stress levels^46^. On the other hand, research about the Iridaceae family's response to salinity stress is minimal since *C. sativus* is almost one of the few medicinal-spice crops of this family, and almost most of the species are ornamental ^47,48^. The effect of salinity stress on the level of photosynthetic pigments has been studied and reported in nearly all families of ornamental plants^49^. Still, no case was reported from Iridaceae. But, electron microscopy observations showed that chloroplast structure under salinity stress in Iris halophila (Iris family) was not seriously damaged^50^. Regarding leaf carotenoid content, this photosynthetic pigment may increase in plants under salinity stress by the induction of ABA hormone through the mevalonic acid pathway^51^.

The amount of chlorophyll degradation is one of the main factors for detecting resistant or non-resistant plants under stress conditions. In many cases, the decrease in leaf area by stress is caused by increasing the chlorophyll concentration in the leaf. However, the changes in the photosynthetic pigment content of saffron were not imposed by the leaf area changes under stress. Thus, it could relate to the tolerance or adaptation mechanism. Resistance plants protect the photosynthetic system; meanwhile, increased photosynthetic efficiency and plant growth were seen in some salinity stress levels^52^.

An increase in chlorophyll content following the application of *F. mosseae* has been reported in plants under salinity stress^53^. According to the results obtained from this research, using jasmonate in stress and non-stress conditions did not make a significant difference in chlorophyll content. However, the maximum effect of + MeJ + AM was on the total chlorophyll content of *C. sativus* in the control treatment and 25 and 50 mM stress levels. Asenio et al.^54^ recorded the highest chlorophyll content with mycorrhiza and jasmonate treatment under control and stress conditions.

This research showed that salinity stress increased the activity of CAT, PPO, and SOD enzymes in *C. sativus*. The activity of enzymatic antioxidants like PPO^55^, CAT^56^, and SOD^11^ increases under salinity stress. Each enzyme has a specific function to reduce stress damage. For example, SOD converts oxygen radical O_2_^−^ to H_2_O_2_, and CAT plays a vital role in salinity stress in converting H_2_O_2_ to water and O_2_ and sweeping oxygen free radicals^57^. The PPO enzyme activity increase under stress is a sign of decomposition and oxidation of toxic compounds due to salinity stress^55^.

On the other hand, PPO generally increases secondary metabolites such as phenols and anthocyanin by stimulating phenylpropanoid pathways^58^. Increasing these antioxidant compounds is another mechanism of plants against stress-induced damage^59^. Jasmonates increase synthesizing secondary metabolites such as anthocyanin and phenolic compounds^60^. Jasmonate induces the synthesis of antioxidant enzymes (PPO and PAL)^61^, which leads to synthesizing non-enzymatic antioxidants like anthocyanins efficiently^22^. It has even been reported that exogenous use of MeJ regulates mRNA associated with these enzymes (PPO and PAL)^62^. The mycorrhizal species of *Glomus intraradices* have been reported to increase PPO activity in maize^63^. This enzyme decreased in the + AM treatments in the current study. Naturally, many of these responses may vary depending on the species of fungi and plants. It was observed that overall, MeJ and mycorrhiza improved the activity of antioxidant enzymes (CAT and SOD) in *C. sativus* at some salinity levels. Indeed, the increasing activity of antioxidant enzymes such as CAT and SOD by *F. mosseae* reduces stress damage and improves growth^44^. Moreover, some studies report the CAT increase by MeJ under salinity stress^18,64^. However, regarding the current research results, this hormone's effect on CAT activity was more evident in higher salinity levels. A similar trend was observed in German chamomile, in which a 75 mM MeJ increased CAT but increased rapidly at high salinity concentrations^19^. Another study on the *Mentha piperita* indicated CAT activity did not change significantly by the MeJ application (in both mycorrhizal plants and non-inoculated) under non-stress conditions and low-stress levels.

The activity of this enzyme was increased in a combination of MeJ and mycorrhizal fungi by increasing salinity level^15^. The present study obtained the same trend in *C. sativus* up to 100 mM. In + MeJ treatments, an increase in SOD was observed in 100 and 125 mM salinity levels, but this phytohormone decreased the activity of this enzyme in the plant in the lower salinity levels. Similar to the results of this research, a study on peppermint reported a decrease in SOD at the control level and low levels of salinity stress by MeJ. However, the activity of this enzyme by MeJ increased significantly under severer stress levels 9. But generally, it was reported that MeJ increases SOD in ad salinity stress^13,64^. The highest SOD activity on most stress levels was observed in + MeJ + AM treatments (Fig. 3B). In another research, SOD activity in the combination of MeJ and *F. mosseae* treatment was higher than the activity of this enzyme in each treatment alone (jasmonate alone or mycorrhiza alone)^65^.

Proline, in addition to the role of osmoregulation under salinity stress, is one of the influential factors in ROS detoxification, protein/enzyme stabilization, and protection of membrane integrity^8^. Its increase was reported under salinity stress in *C. sativus*^66^ and several other plants of the Iridaceae family, such as *Iris hexagonal*^67^ and *Iris lactea* Pall^68^. It was claimed that MeJ influences directly or by other phytohormones, such as ABA, on proline levels in salinity conditions^69^. Moreover, the improvement of proline levels has been reported in salinity stress conditions by MeJ in many plants, including olives^70^, chamomile^19^, and soybeans^13^. In line with the current study, it was reported TSS increased by exogenous use of jasmonate^71^. But salinity had no significant effect on this osmolyte of *C. sativus* leaves. This plant does not change the amount of TSS to regulate the osmotic potential of its cells. Studies have shown the amount of soluble sugar in the leaves of *C. sativus*^72^ and leaves of *Iris halophile*^72^ did not change significantly in many levels of salinity stress. Moreover, it was observed that the soluble sugar content of *C. sativus* under drought stress did not change in the first trimester (equation approximately 120 days)^73^. Hence, these responses may relate to this family's physiological behavior or at least this plant.

The corm of *C. sativus* has one main bud in the center and several lateral ones on the mother corm. Flowers and leaves are produced from the buds after planting. However, the central bud has more leaf area (leaf size and number) than the lateral bud^68^. Based on the results from Fig. 5, the role of the main bud in producing the total leaf area was minimally 60%. Decreased leaf area as an energy production source is one of the most critical factors that limit plant growth under stress. Stress significantly damages plants, but according to the results of this research, the destruction of the photosynthetic system was not a limiting factor in the growth of this plant. One of the main limiting growth factors is probably the changes in leaf area under severe stress levels because, regarding the leaf structure, *C. sativus* has a limited leaf area compared to other plants. So 29% reduction in leaf area under 125 mM damages the photosynthetic efficiency of this plant significantly. Salinity stress limits the photosynthesis system by impairing stomatal function, disrupting the system of nutrient uptake and osmotic balance, and allocating a significant amount of energy to deal with stress's damage which finally influences the plant growth and leaf area^71^.

On the other hand, the development of daughter corms on the mother corm continues throughout vegetative growth. In this period, organic compounds are transferred from the mother corm to the daughter corm in addition to current photosynthesis. Eventually, the mother corm decays over time until it is destroyed at the end of the growth period^74^. The present research results showed that the treatments, including stress, did not influence the rate of nutrients and organic compounds transfer from the mother corm to the daughter because the changes in the dry weight of the mother corm under stress were not significant. Moreover, changes in the dry weight of contractile roots were not substantial. The contractile root is not used in stress for the plant. Mycorrhiza improves plant growth and reduces salinity stress damage through the enzymatic and non-enzymatic defense, osmolytes content, and increased nutrient uptake ^75–80^.

The use of jasmonate increased plant growth alone at many stress levels. One reason for improving plant growth, total and corm dry weight at different stress levels, especially high-stress levels such as 100 mM, by jasmonate was the effect of this phytohormone on the concentration of osmolytes, enzymatic activities (such as CAT and SOD and PPO), and non-enzymatic antioxidants (anthocyanins and carotenoids)^13,14^. Based on the results of this research, it cannot be claimed that the MeJ effect is not always positive on the plant because it significantly reduced proline, CAT, and total dry weight at 125 mM. Moreover, the combination of jasmonate and mycorrhiza caused MD to be negative at the 125 mM level.

## Conclusion

Generally, low salt concentrations (about 25 mM) in the root environment improved *C. sativus* growth. Growth and development of *C. sativus* were substantially impaired at 100 and 125 mM salinity stress levels. The effect of stress on the mother corm's weight was insignificant. Therefore, stress conditions do not occur any limitations on the absorption of the organic compounds by the daughter corm from the mother corm. Also, damaging photosynthesis in salinity stress conditions was not the limiting factor of the plant's growth. However, considering that *C. sativus* has a limited leaf area, the decrease in leaf area at severe stress levels can be regarded as the leading cause of damage. Generally, using jasmonate and mycorrhiza improves plant growth in stress conditions (mainly at 50, 75, and 100 mM) by improving enzymatic and non-enzymatic antioxidants and increasing levels of osmolytes. However, combining these two factors damaged the plant under125 mM stress salinity level.

## Data Availability

All the data are embedded in the manuscript.
