# Author Correction: Co-application of Mycorrhiza and methyl jasmonate regulates morpho-physiological and antioxidant responses of *Crocus sativus* (Saffron) under salinity stress conditions

**DOI:** 10.1038/s41598-023-35118-3

**Published:** 2023-05-17

**Authors:** Mohammad Hamidian, Mohsen Movahhedi-Dehnavi, R. Z. Sayyed, Waleed Hassan Almalki, Abdul Gafur, Bahman Fazeli-Nasab

**Affiliations:** 1 Department of Agronomy and Plant Breeding, Faculty of Agriculture, Yasouj University, Yasouj, Iran; 2 Department of Microbiology, PSGVP Mandal’s S I Patil Arts, G B Patel Science and STKV Sangh Commerce College, Shahada, 425409 India; 3 Department of Pharmacology, College of Pharmacy, Umm Al-Qura University, Makkah, 24382 Saudi Arabia; 4 Sinarmas Forestry Corporate Research and Development, Perawang, Indonesia; 5 Department of Agronomy and Plant Breeding, Agriculture Institute, Research Institute of Zabol, Zabol, Iran; 6 Plant Biotechnology and Breeding Department, College of Agriculture, Ferdowsi University of Mashhad, Mashhad, Iran

Correction to: *Scientific Reports* https://doi.org/10.1038/s41598-023-34359-6, published online 06 May 2023.

The original version of this Article contained an error in the title of the paper, where the term “co-application” was incorrectly given as “co-inoculation”.

In addition, the Article contained an error in the Acknowledgments section.

“The authors thank Yasouj University, Iran, for providing the necessary material and facilities to perform this work and the Deanship of Scientific Research at Umm Al-Qura University, Makkah, Saudi Arabia, for supporting this work by Grant Code (Project Code: 22UQU4310387DSR12). The research fellowship granted by the Alexander von Humboldt Foundation, Bonn, Germany, to AG is also gratefully acknowledged.”

Now reads:

“The authors thank Yasouj University, Iran, for providing the necessary material and facilities to perform this work and the Deanship of Scientific Research at Umm Al-Qura University, Makkah, Saudi Arabia, for supporting this work by Grant Code (Project Code: 22UQU4310387DSR002). The research fellowship granted by the Alexander von Humboldt Foundation, Bonn, Germany, to AG, is also gratefully acknowledged.”

The original Article has been corrected.

